# Tb II-I, a Fraction Isolated from *Tityus bahiensis* Scorpion Venom, Alters Cytokines’: Level and Induces Seizures When Intrahippocampally Injected in Rats

**DOI:** 10.3390/toxins10060250

**Published:** 2018-06-19

**Authors:** Emidio Beraldo Neto, Douglas O. C. Mariano, Lucas A. Freitas, Ana L. C. Dorce, Adriana N. Martins, Daniel C. Pimenta, Fernanda C. V. Portaro, Daniela Cajado-Carvalho, Valquiria A. C. Dorce, Ana L. A. Nencioni

**Affiliations:** 1 Laboratory of Pharmacology, Butantan Institute, Av. Dr. Vital Brazil 1500, São Paulo 05503-900, Brazil; emidio.beraldo@gmail.com (E.B.N.); lucas.freitas@buantan.gov.br (L.A.F.); leticia_bio@yahoo.com.br (A.L.C.D.); adriana.martins@butantan.gov.br (A.N.M.); valquiria.dorce@butantan.gov.br (V.A.C.D.); 2 Laboratory of Biochemistry and Biophysics, Butantan Institute, Av. Dr. Vital Brazil 1500, São Paulo 05503-900, Brazil; douglas.mariano@butantan.gov.br (D.O.C.M.); dcpimenta@butantan.gov.br (D.C.P.); 3 Laboratory of Immunochemistry, Butantan Institute, Av. Dr. Vital Brazil 1500, São Paulo 05503-900, Brazil; fernanda.portaro@butantan.gov.br (F.C.V.P.); daniela.carvalho@butantan.gov.br (D.C.-C.)

**Keywords:** scorpion venom, neurotoxins, hippocampus, behavior, cytokines

## Abstract

Scorpion venoms are composed of several substances with different pharmacological activities. Neurotoxins exert their effects by targeting ion channels resulting in toxic effects to mammals, insects and crustaceans. Tb II-I, a fraction isolated from *Tityus bahiensis* scorpion venom, was investigated for its ability to induce neurological and immune-inflammatory effects. Two putative β-sodium channel toxins were identified in this fraction, Tb2 II and Tb 4, the latter having been completely sequenced by mass spectrometry. Male Wistar rats, stereotaxically implanted with intrahippocampal cannulas and electrodes, were injected with Tb II-I (2 µg/2 µL) via the intrahippocampal route. The behavior, electrographic activity and cellular integrity of the animals were analyzed and the intracerebral level of cytokines determined. Tb II-I injection induced seizures and damage in the hippocampus. These alterations were correlated with the changes in the level of the cytokines tumoral necrosis factor-α (TNF-α) and interleukin-6 (IL-6). Therefore, the binding of Tb II-I to its target in the central nervous system may induce inflammation resulting in neuropathological and behavioral alterations.

## 1. Introduction

Scorpion venoms are a mixture of substances such as peptides, enzymes, mucoproteins, amines, inorganic salts, lipids, protease inhibitors and neurotoxins, among others [1,2,3]. The major components of scorpion venoms are neurotoxins, disulfide-bridged peptides characterized by a three-dimensional structure that is comprised of an α-helix and three segments of anti-parallel β-sheet structures [4]. Due to their interaction with ion channels, neurotoxins cause massive release of neurotransmitters by affecting the permeability of excitable membranes [3,5,6]. The outcome of this release is the stimulation of sympathetic and parasympathetic systems, which reflects the common symptoms observed in scorpion envenomation, such as tachycardia, hypertension, hyperglycemia, sweating, bradycardia, hypotension, secretions, in addition to central stimulation causing hyperthermia, vomiting and convulsion [1,7].

Moreover, evidence from animal studies and clinical cases has demonstrated the involvement of the inflammatory cascade and release of cytokines in the pathogenesis of many envenomation syndromes [7,8,9,10,11,12]. However, these studies refer only to systemic cytokine release.

It is uncertain if scorpion toxins cause inflammation in the central nervous system (CNS) in the same way as in peripheral nerves and what would be the consequences. Few studies are available regarding the inflammatory action of venoms or toxins in the CNS. Recently, it was demonstrated that the intracerebral injection of TsTX, a toxin isolated from *Tityus serrulatus* scorpion venom, caused an increase in the hippocampal level of interferon-γ (INF-γ) in rats, concomitantly with behavioral and electrographic alterations [13]. In addition, when this toxin was subcutaneously injected in rats, it caused an increase in leukocyte rolling and adhesion and high levels of tumoral necrosis factor-α (TNF-α) in the brain [14]. Moreover, the intracerebroventricular injection of KTX_2_, a toxin isolated from *Androctonus australis*, caused an inflammatory response in the brain of mice and had consequences on pancreatic and hepatic functions, probably mediated by the release of interleukin-6 (IL-6) and TNF-α [15].

Cytokines are important mediators in the CNS and are expressed in normal adult brain [16]. They act as neuromodulators in addition to their pro-inflammatory action, particularly interleukin-1 (IL-1) and TNF-α [16,17]. However, most studies relate cytokines to CNS pathologies. Notably, cytokines play a substantial role in the development of the epileptogenic process [17], since the neuronal excitability is increased and the seizure threshold is decreased by brain inflammation [18].

It is known that convulsion often occurs in severe cases of scorpion envenomation [1,7,19,20], and its origin has been mainly related to systemic effects of the venom, altering the release of sympathetic and parasympathetic neurotransmitters [1]. It has also been demonstrated that central neurotransmitters, mainly glutamate, participate in this process (revised by [21]). However, the participation of cytokines has not been taken into account.

Thus, the aim of the present study was to determine the neurological and immunological effects of Tb II-I, a fraction obtained from the venom of *T. bahiensis*, one of the main scorpions responsible for severe accidents in Brazil [22]. The hippocampal way was chosen because, as part of the limbic system, it is one of the brain areas most affected in epileptogenic processes [23].

We found that Tb II-I causes electrographic and behavioral epileptiform changes when injected into the hippocampus, along with neuronal loss and an increase in the level of TNF-α and IL-6, particularly important cytokines in convulsive processes.

## 2. Results

### 2.1. Chromatography, Biochemical Characterization and Mass Spectrometry Analysis of Tb II-I

**Figure 1:**
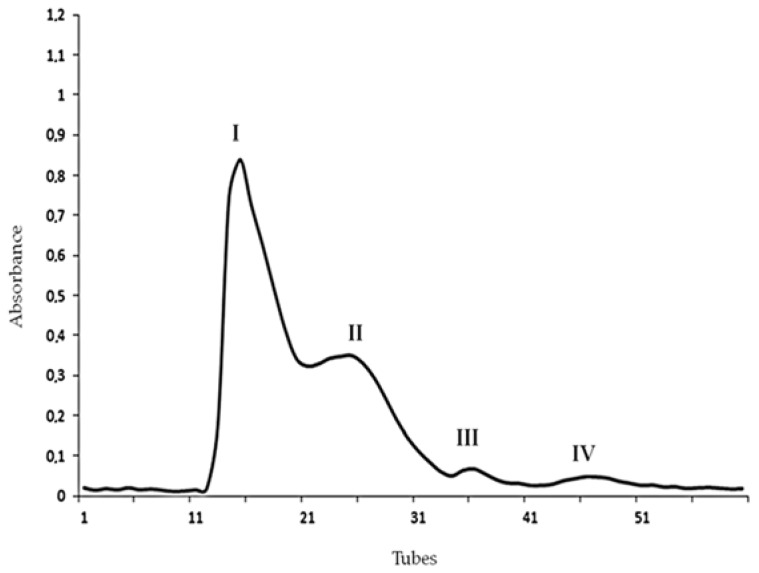
Size-exclusion chromatography (SEC) profile of *Tityus bahiensis* scorpion venom, showing four semi-purified fractions (pools) designated I–IV.

Initially, *T. bahiensis* venom was analyzed by size-exclusion chromatography (SEC), in which four fractions, designated I, II, III and IV, were obtained, as illustrated in Figure 1.

The intrahippocampal injection of Fractions I and II (2 µg/µL) evoked behavioral and electrographic alterations, as illustrated in Table 1. Fractions III and IV (2 µg/µL) caused only prostration in some animals and did not affect the electrographic activity of the animals (Table 1). The injection of Ringer’s solution (1 µL) did not cause any alteration.

**Figure 2:**
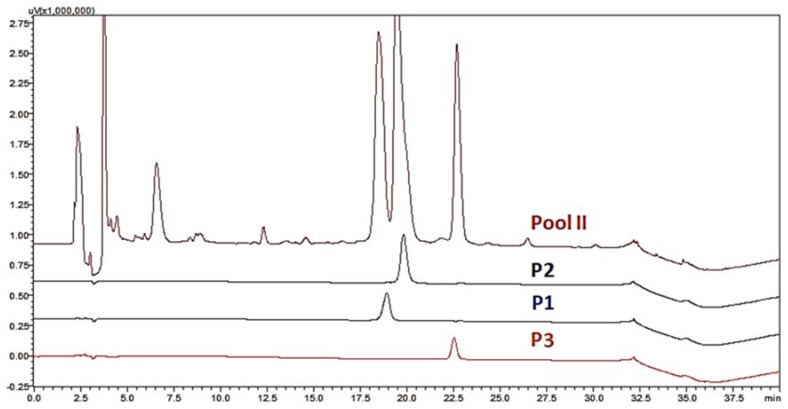
High-performance liquid chromatography (HPLC) of Fraction II derived from SEC of *Tityus bahiensis* scorpion venom. The three collected peaks were designated Tb II-I (p1), Tb II-II (p2) and Tb II-III (p3).

As Fraction II exhibited intense neurological effects, it was analyzed by RP-HPLC. Three peaks were collected and designated Tb II-I, Tb II-II and Tb II-III (Figure 2). Once Tb II-II and Tb II-III revealed a complex RP-HPLC profile (for more details, see Supplementary Figure S1), Tb II-I was selected for further processing and biological characterization.

**Figure 3:**
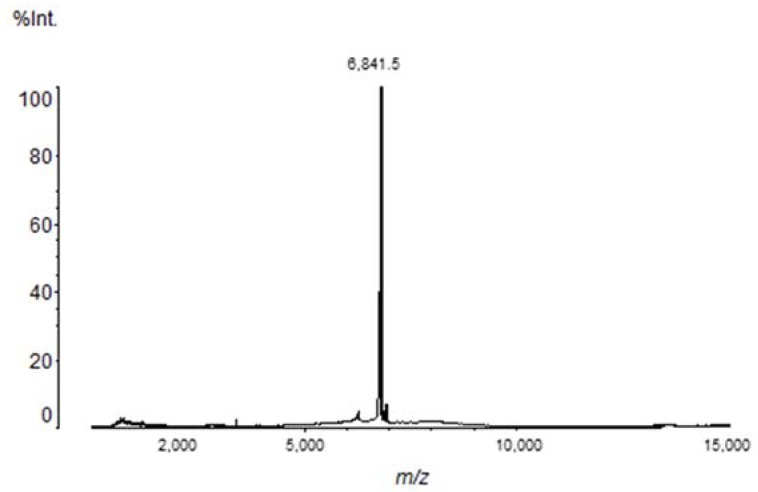
MALDI-TOF/MS profile of Tb II-I. It is possible to observe the presence of a major peptide (6.84 kDa).

Matrix-assisted laser desorption ionization-time of flight (MALDI-TOF) analysis indicated the presence of a major component with a molecular mass of 6842.40 Da (Figure 3). Tb II-I N-terminal sequencing was obtained by Edman degradation and the first nine amino acids were determined: GKEGYPTDK.

**Figure 4:**
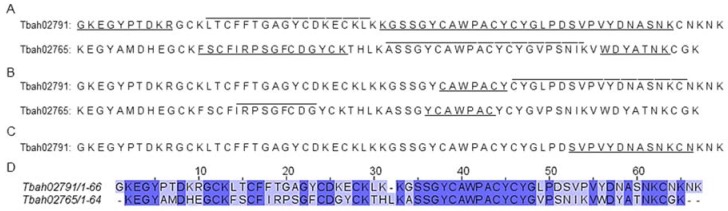
Protein sequences of two toxins identified in Tb II-I. This fraction was submitted to in solution digestion by (**A**) trypsin, (**B**) chymotrypsin or (**C**) formic acid, and the peptidomic analysis was performed against a transcriptome database obtained from the venom gland of *Tityus bahiensis*. The identified peptides for each protein are highlighted. Two beta sodium toxins Tbah02791 (known as Toxin Tb4; UniProt code: P56610) and Tbah02765 (known as Toxin Tb2 II; UniProt code: P60276) were identified. (**D**) Tbah02791 and Tbah02765 Clustal O (1.2.4) sequence alignment analysis, showing that both sequences have 56.72% identity.

Furthermore, a chemically- or enzymatically-proteomic approach was performed, and the results were analyzed against a transcriptome database obtained from the venom gland of *Tityus bahiensis*. We found the presence of two toxins in Tb II-I, Tbah02765 (known as Toxin Tb2 II-UniProt code: P60276) and Tbah02791 (known as Toxin Tb4; UniProt code: P56610) (Figure 4). To check the identified peptides, see Supplementary Figures S2–S6 and Table S1. Both toxins have 56.72% similarity between each other (Figure 4D).

**Figure 5:**
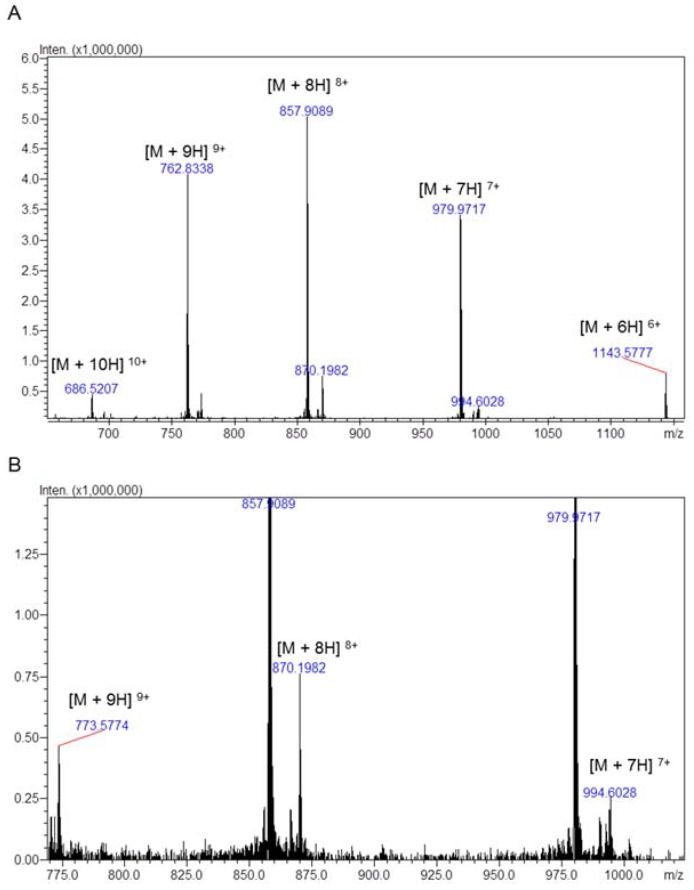
Direct infusion ESI-IT-TOF/MS profile of Tb II-I. The multiple charge ions are indicated and above each its *m*/*z* value. The calculated molecular masses are (**A**) (6851.71 ± 0.91) Da for the major toxin and (**B**) (6950.30 ± 0.98) Da for the second toxin.

Toxin Tb2 II was characterized by Pimenta [24], and Toxin Tb4 was partially described (only the first 19 amino acids) by Becerril [25]. Our results suggest that toxin Tb4 is composed by 63 amino acids, since the C-terminal sequenced by mass spectrometry was: GLPDSVPVYDNASNKCN or SVPVYDNASNKCN (Figure 4B,C); and the experimental masses obtained for Tb4 and Tb2 II were 6851.71 Da and 6950.30 Da, respectively (Figure 5, Table 2).

### 2.2. Electroencephalographic Recording and Behavioral Observation

The intrahippocampal injection of Tb II-I (2 µg/2 µL) evoked electrographic alterations such as slow waves (33% of animals) and strong discharges in the cortex and hippocampus (67% of animals), as well as grouped spikes (67% of animals) only in the hippocampus. These alterations started approximately 40 min after the injection and persisted for about 2–3 h (Table 3). The control animals injected with Ringer’s solution (2 µL) did not display any alteration (Table 3).

Behavioral alterations such as salivary and lachrymal secretions (33%), “wet dog shakes” (WDS) (67%), penile erection (50%) and myoclonus (33%) were observed in the experimental animals (Table 3). No changes in behavior occurred in the control animals (Table 3).

### 2.3. Histopathological Analysis

**Figure 6:**
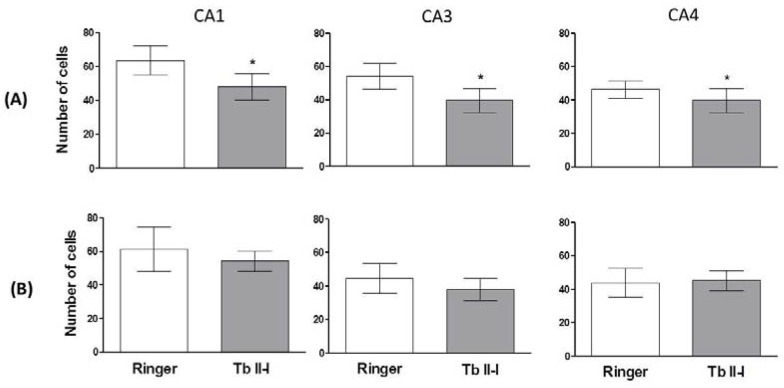
Histological sections of rat brains (10 μm) analyzed by light microscopy at 400×. The analysis refers to the layer of (**A**) injected with Tb II-I (2 µg/2 µL) or Ringer’s solution (2 µL) and the (**B**) non-injected side of the hippocampal areas CA1, CA3 and CA4. Data are expressed as the mean ± SD. * *p* < 0.05 compared to the control group (Student’s *t*-test).

A decrease in the number of pyramidal neurons in CA1, CA3 and CA4 ipsilateral (side of injection) areas was observed after the intrahippocampal injection of the Tb II-I. No changes were observed in the contralateral areas of the hippocampus after injection of Tb II-I (Figure 6), and no changes in ipsilateral and contralateral areas of the hippocampus were observed in control animals after injection of Ringer’s solution (Figure 6).

### 2.4. Cytokine Levels

**Figure 7:**
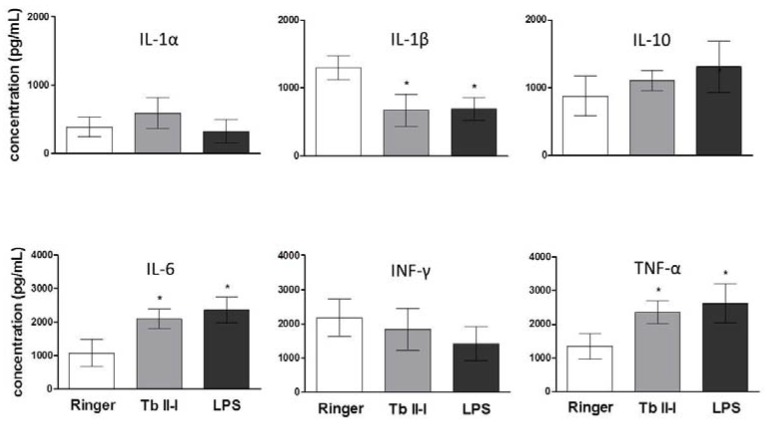
Cytokine levels in the hippocampus of rats 6 h after the administration of Tb II-I (2 µg/2 µL), Ringer’s solution (2 µL) or LPS (10 µg/µL). Data are expressed as the mean ± SD. * *p* < 0.05 compared to the control group (Analysis of Variance (ANOVA) followed by Tukey’s test).

There was an increase in the intrahippocampal levels of IL-6 and TNF-α, while the level of interleukin-1β (IL-1β) was decreased (Figure 7). The same changes were observed in the animals treated with lipopolysaccharide (LPS) (Figure 7).

## 3. Discussion

The release of neurotransmitters is affected by the action of many scorpion toxins that interact with ion channels [6]. Several experimental studies have already demonstrated epileptiform activity and neuronal loss in animals intracerebrally injected with scorpion toxins [26,27,28], mainly due to the excessive release of glutamate [13,29]. On the other hand, cytokines are also involved in convulsive processes, particularly IL-1, IL-6 and TNF-α [30,31,32].

In cases of scorpion envenomation, the increase in the systemic level of cytokines such as IL-1β, IL-6, IL-8, IL-10 and TNF-α has been widely demonstrated [11,33]. This increase was also observed in vitro in macrophages after exposure to *T. serrulatus* venom, showing changes in the levels of IL-1α, IL-β, IL-10, INF-γ and TNF [7,34]. However, few studies have been conducted regarding alteration in cytokine levels in the brain in accidental or experimental cases of envenomation and the possible consequences [13,14]. Not neglecting the importance of neurotoxins and their effect on ion channels in scorpion envenomation, increased cytokines also can be related to different toxicological manifestations, for example convulsion and excitotoxicity. Therefore, in the present study, we performed experiments to better understand the pathophysiological and immune-inflammatory effects on the hippocampus that can be caused by severe scorpion envenomation, using Tb II-I isolated from the venom of *T. bahiensis*.

Transcriptome analysis from venom gland of *Tityus bahiensis* showed that 8.1% and 12.9% of toxin transcripts codify a potassium or sodium channel toxin, respectively [35]. Our peptidomic analyses, after being analyzed against a transcriptome database, identified two toxins in Tb II-I: Tbah02765 and Tbah02791. Both toxins were described by de Oliveira [25] as putative β-sodium channel toxins, based on their sequence similarity with known toxins.

Tbah02765 was previously isolated and characterized by Pimenta [24], being called Tb 2 II. This toxin showed lethality (>50% of injected animals dead) in mammals (100 ng/mouse, after intracerebroventricular injection) and house flies or a toxic effect (<50% of injected animals dead) in crickets and cockroaches. Tbah02791 has 100% identity to the Tb4 (or TbTX-VI) toxin described by Becerril [26], who only sequenced the first 19 amino acids. Tb4 was not toxic in mice after intraperitoneal injection (assayed with 50 µg/animal) [25].

In our work, it was possible to completely sequence the Tb4 toxin, by mass spectrometry analyses. Two approaches were used to sequence the C-terminal: chymotrypsin or acetic acid cleavage (Figure 5B,C); as we could not identify the last three amino acids (KNK) for the toxin Tb4, we suggest that these three amino acids may be removed during protein maturation or activation. Furthermore, the experimental mass obtained for Tb4 was 6851.71 Da, close to the theoretical mass of 6851.04 Da.

Cytokines are physiologically present in the adult brain at relatively low levels under physiological conditions, and they act not only as inflammatory mediators, but also as neuromodulators [16,32], supporting the development and function of the nervous system [36]. Both glia and neurons release cytokines and express their receptors [32], and they occur mostly in microglia and astrocytes in the CNS [32]. Dysregulation of the synthesis, release or cell signaling of cytokines can contribute to neuronal dysfunctions [32]. The excitability of neurons is rapidly altered by the activation of cytokine receptors by modifying the function of voltage-gated and receptor-coupled ion channels [32]. Inflammatory cytokines can affect monoamine neurotransmitters by decreasing their synthesis and release [37].

In our experiments, an epileptogenic behavior accompanied by neuronal loss and increased levels of inflammatory cytokines such as IL-6 and TNF-α after intrahippocampal injection of Tb II-I was observed. The inflammation process has a role in the molecular and structural alterations responsible for seizures and epilepsy in humans [30,32,38] and in animal models [39]. TNF-α, IL-6 and IL-1 are among the major cytokines involved in epileptic processes [30]. It seems that Tb II-I used in the present study has an inflammatory effect, since it affected the level of cytokines in a similar fashion as the injection of LPS, an agent widely used in neuroinflammation models [40]. The activation of innate immune responses of glial cells, thereby inducing epileptic seizures and hippocampal sclerosis, was demonstrated in rats after the hippocampal infusion of LPS [41].

In our study, we observed a substantial increase in TNF-α level in the hippocampus. This cytokine is present in the brain [42], where it exerts both homeostatic and pathophysiological roles [43]. Under physiological conditions, it maintains normal synaptic functions, but it produces adverse effects in pathological conditions, when large amounts of TNF-α are released by astrocytes and mainly by microglia, potentiating glutamate-mediated excitotoxicity [42,43]. It has been demonstrated that TNF-α acts directly on neurons, increasing the surface expression of α-amino-3-hydroxy-5-methyl-4-isoxazolepropionic acid (AMPA) glutamatergic receptors (AMPARs) [44,45]. Most of these receptors lack the GluR2 subunit, which makes them calcium permeable [45]. AMPARs are responsible for fast excitatory synaptic transmission [42], and their activation is necessary to relieve the blockage of NMDA receptors. Once unblocked, *N*-methyl-d-aspartate (NMDA) receptors become calcium permeable, and excessive intracellular calcium triggers the excitotoxic process [42]. Therefore, excitotoxicity can be determined both directly and indirectly by abnormal trafficking of AMPARs [42]. Moreover, TNF-α increases the endocytosis of GABA_A_ receptors, reducing their surface expression [45]. The fast inhibitory synaptic transmission in the CNS is determined mainly by GABA_A_ receptors, being extremely important in the regulation of neuronal networks [46]. The consequence is an imbalance between excitation and inhibition, having important implications for the role of TNF-α in neuropathology [45]. Finally, TNF inhibits glial neuronal glutamate transporters on astrocytes [47], resulting in the increase of glutamate concentration in the parenchyma and leading to excitotoxicity [43]. Accordingly, the increase in TNF-α concentration caused by Tb II-I could be, at least in part, responsible for the seizures and epileptiform behavior observed in the experimental animals, as well as for the neuronal loss. Our results agree with data from the literature that suggest that inflammation in the hippocampus, caused predominantly by TNF-α signaling, contributes to hyperexcitability and acute seizure in a mouse model of limbic epilepsy [48].

IL-6 also increased significantly. IL-6 is an important cytokine, required for normal development of the nervous system [39,49,50], that can regulate neuronal and synaptic function and behavior [49]. Under physiological conditions, low levels of IL-6 are found in the brain [50] and are produced in the CNS mainly by astrocytes. However, microglia and neurons are also an important source of IL-6 [49,51]. The IL-6 receptor system is expressed in many regions of the CNS, both in neurons and glia [49]. IL-6 can interact with two forms of receptor, a specific transmembrane receptor responsible for classical signaling and a soluble form responsible for trans-signaling [49,50], each one having a specific role, protective or neurodegenerative, respectively [50]. The effects of IL-6 in the CNS are controversial, and results from clinical studies and measured values of IL-6 are often inconsistent [49]. It has been reported that IL-6 seems to have neuroprotective effects, while others suggest its involvement in excitotoxicity-induced brain damage. Many studies have demonstrated the neuroprotective effects of IL-6 in ischemic brain injuries, although few studies suggest that IL-6 has an injurious effect [52]. Numerous neurological disorders, such as multiple sclerosis and Parkinson’s and Alzheimer’s disease, are characterized by elevated levels of IL-6 in the CNS [49,50]. An increased level of IL-6 in the brain has been associated with neurotoxic and pro-convulsive effects [39] and with altered cognitive function and behavior [49]. Intracerebral administration of IL-6 in rats has been demonstrated to induce status epilepticus, decreasing GABAergic inhibition through a decrease in receptor density, without changing glutamatergic excitation, resulting in an imbalance between inhibitory and excitatory inputs, shifting toward excitation [53]. On the other hand, it was demonstrated that IL-6 has a neuroprotective action against ischemia and glutamate excitotoxicity and inhibits the spread of excitation, inhibiting the depolarization-evoked glutamate release from neocortical synaptosomes [54]. IL-6 has been found to increase neuronal survival in a variety of experimental conditions [54]. It is uncertain whether the increase in the level of IL-6 observed in our study is harmful or protective. It could contribute to the intensification of the convulsive and toxic process. Conversely, it could be an attempt to protect against the effects caused by TNF-α. Further studies are necessary to better understand the process.

IL-1 is constitutively expressed at very low levels in the CNS of humans and rodents [39,55] and is crucial for normal synaptic functioning [56]. On the other hand, IL-1β has been shown to be involved in the modulation of some neurological dysfunctions [57]. Several animal experiments have demonstrated the epileptogenic potential of high IL-1β concentration [39], and the administration of the naturally-occurring antagonist of IL-1 is able to inhibit motor and electroencephalographic seizures [58]. IL-1β affects various classical neurotransmitter systems, and there is evidence that the NMDA glutamate receptor and IL-1β interact with each other. For example, when NMDA is injected in the brain of rats in the postnatal period, the production of IL-1β is stimulated [59] or when antagonists of the NMDA receptor are injected in rats, the proconvulsant action of IL-1β is blocked [60]. The NMDA receptor function is increased by IL-1β through activation of tyrosine kinase and phosphorylation of the NR2A/B subunit, contributing to neurodegeneration evoked by glutamate [61]. Increased levels of IL-1β and its receptor (IL-1R1) were found in samples from patients with temporal lobe epilepsy, and this increase was correlated with the frequency of seizures [62]. Curiously, in our experimental animals, we observed a decrease in the level of IL-1β, although it plays an important role in the inflammatory process and is a hallmark of seizures [63]. In our study, it was presumed that this decrease might have been a rebound effect, as a response to an increase in the level of IL-1β in the first hours after injection of Tb II-I, followed by a decrease in the subsequent hours. This was similarly observed previously by Minami [64], who demonstrated an increase in IL-1β mRNA expression 2 h after intraperitoneal injection of kainic acid, followed by a decline after 4 h. A similar effect was observed after intrahippocampal injection of a toxin isolated from *Tityus serrulatus* venom [13], suggesting that these toxins trigger a quick response.

## 4. Conclusions

In conclusion, it was demonstrated that the scorpion toxins present in Tb II-I can induce an inflammatory process also in the CNS, and the imbalance in cytokine levels may be responsible, at least in part, for the behavioral and electroencephalographic alterations observed in the animals. Other systems and mediators may be involved in the process, possibly neurotransmitters, and this issue needs further investigation. It is important to understand the central effects of toxins not only to determine their participation in the envenomation process, but also to use them as pharmacological tools in the study of the CNS and the pathologies related to the immune inflammatory processes.

## 5. Materials and Methods

### 5.1. Venom Fractionation

*T. bahiensis* lyophilized whole venom was extracted from scorpions maintained in captivity at the Arthropod Laboratory of the Butantan Institute by electrical stimulation of the telson and manually collected in amber microtubes, provided by the Strategic Nucleus of Venoms and Antivenoms, São Paulo, Brazil. Twenty five milligrams of venom were dissolved in 500 μL of sodium chloride (35 mM) and ammonium acetate (30 mM) solution, with pH adjusted to 5.5 with acetic acid. The reagents were purchased from Merck (Darmstadt, Germany). The venom sample was homogenized, centrifuged (14,170× *g*, 4 °C) for 8 min and the supernatant stored at 4 °C. The precipitate was resuspended in the same buffer (500 µL), homogenized and centrifuged. This procedure was repeated 3 times, and supernatants were gathered to the first one, finalizing with a solubilized venom sample of 2.0 mL. The venom sample was applied to a Sephadex (G25 fine) column (GE Healthcare Bio-Sciences AB, Uppsala, Sweden) (1.6 cm in diameter and 70 cm in height), and fractions were collected using a Bio-Rad automatic fraction collector (Bio-Rad, Hercules, CA, USA) after discarding the extra volume (±25 mL). The chromatography was carried out under a flow rate of 3.6 mL/h, and four fractions (1.5 mL each) were obtained, based on their optical density monitored spectrophotometrically at 280 nm. The fractions were lyophilized and stored at −20 °C until use.

The fraction containing Tb II-I was suspended in 1.5 mL of water and subjected to a reverse-phase high-performance liquid chromatography (RP-HPLC) system (Prominence, Shimadzu Co, Kyoto, Japan) using a Restek RP-C18 analytical column (5 μm, 250 mm × 4.6 mm). The column was eluted at a constant flow rate of 1.0 mL/min, with a 25–37.5% of Solvent B over 25 min (Solvent A: H_2_O/0.1% trifluoroacetic acid (TFA); Solvent B: acetonitrile +10% Solvent A), and the eluate was monitored at 214 nm. The reagents were purchased from JT Baker (Xalostic, Mexico). All RP-HPLC fractions were freeze-dried and stored at −20 °C until use.

### 5.2. Mass Spectrometry

The RP-HPLC fraction was analyzed using a matrix-assisted laser desorption ionization-time of flight (MALDI-TOF) mass spectrometer (Axima Performance, Shimadzu). One microliter of the sample was co-crystallized with 1 µL of α-cyano-4-hydroxycinnamic acid matrix (saturated solution prepared in 50% ACN/0.1% TFA) in the plate and dried at room temperature. The mass spectrum was obtained in the 50–15,000 mass/charge (*m*/*z*) range, in linear positive mode.

Tb II–I was also analyzed in an electrospray-ion trap-time of flight (ESI-IT-TOF) (Shimadzu Co., Kyoto, Japan) with binary ultra-fast liquid chromatography system (UFLC) (20A Prominence, Shimadzu). The sample was injected using an auto-injector module (SIL-20AC, Shimadzu), and analysis was performed in positive mode (ESI+), at a flow rate of 200 μL/min of 50% Solvent B (Solvent A: water/acetic acid (999/1, *v*/*v*); Solvent B: acetonitrile/water/acetic acid (900/99/1, *v*/*v*/*v*)). The interface voltage used was 4.5, and the detector voltage was 1.85 KV, with a temperature of 200 °C. Mass spectrometry (MS) spectra were collected in the 400–1400 mass/charge rate (*m*/*z*). The data were analyzed by LabSolutions software (LCMSsolution Version 3.60.361, Shimadzu).

### 5.3. Amino Acid Sequencing

The analysis of the N-terminal sequence was performed on a SHIMADZU automatic protein sequencer (PPSQ-10 System) by the Edman degradation method [65]. Three hundred micrograms were used for the analysis. The identified amino acids were separated by HPLC, and quantification and identification were performed by comparison with a 25-pmol standard (analyzed at the beginning of each sequence). The sequences were compared and aligned by means of the BLAST-NCBI algorithm.

### 5.4. In-Solution Digestion and Proteomic Analysis

An aliquot of Tb II-I was dried, resuspended with 8 M urea (in 100 mM Tris-HCL, pH 8.5) and Tris (2-carboxyethyl) phosphine hydrochloride (TCEP) (dissolved in water) (20 mM final concentration) for 1 h, at room temperature; then, iodoacetic acid (IAA) (dissolved in water) (10 mM final concentration) was added and incubated for 1 h, at room temperature and protected from light. Next, 100 mM Tris-HCl (pH 8.5), to dilute the urea concentration to 2 M, and 10 µL of trypsin (10 ng·µL^−1^ in 100 mM Tris-HCl, pH 8.5) or chymotrypsin (50 ng·µL^−1^ in 100 mM ammonium bicarbonate, pH 8.5) were added. The incubation was performed overnight, at 30 °C. The reaction was stopped adding 50% ACN/5% TFA and dried. Besides that, Tb II-I was chemically cleaved according to de the protocol described by Oliveira [66], using formic acid. All the reagents were purchased from Sigma-Aldrich (St. Louis, MO, USA).

Samples were lyophilized, resuspended in 0.1% acetic acid and analyzed by liquid chromatography-mass spectrometry using an ESI-IT-TOF system coupled to binary an ultra-fast liquid chromatography system (UFLC) (20A Prominence, Shimadzu). Each sample was loaded in a C18 column (Discovery C18, 5 μm; 50 × 2.1 mm) in a binary solvent system: (A) water:acetic acid (999:1, *v*:*v*) and (B) ACN: water:acetic acid (900:99:1, *v*:*v*:*v*). The column was eluted at a constant flow rate of 0.2 mL·min^−1^ with a 0–40% gradient of Solvent B over 35 min. The eluates were monitored by a Shimadzu SPD-M20A PDA detector before introduction into the mass spectrometer. The interface voltage was 4.5 KV; the capillary voltage was 1.85 KV, at 200 °C; and the fragmentation was induced by argon collision, at 55% ‘energy’. MS spectra were acquired under positive mode and collected in the 350–1400 mass/charge (*m*/*z*) range. MS/MS spectra were collected in the 50–1950 *m*/*z* range.

LCD Shimadzu raw data were converted (LCMS Protein Postrun, Shimadzu) to Mascot Generic Format (MGF) files prior to analyses. Peaks Studio V7.0 (BSI, Toronto, ON, Canada) was used for data processing (de novo peptide sequencing and proteomic identification) [67]. Peaks Studio V7.0 (BSI, Toronto, ON, Canada) was used for data processing [67]. Proteomic identification was performed according to the following parameters: error mass (MS and MS/MS) set to 0.2 Da; methionine oxidation and carbamidomethylation as variable and fixed modification, respectively; trypsin, chymotrypsin or formic acid as the cleavage method; maximum missed cleavages (3), maximum variable Post-Translational Modifications (PTMs) per peptide (3) and non-specific cleavage (one). Tb II-I was analyzed against a transcriptome database (9151 search entry) obtained from the venom gland of *Tityus bahiensis* (GenBank: GBXR00000000.1) [35].

### 5.5. Animals

Male Wistar rats, 260–280 g, were obtained from the Central Animal Facility of the Butantan Institute and housed in the Laboratory of Pharmacology for the in vivo assays. The animals had ad libitum access to water and food and were maintained under controlled conditions (12 h light/dark cycles at 22 ± 2 °C). All experimental procedures were in accordance with the ethical principles in animal research adopted by the Brazilian Society of Animal Science and the National Brazilian Legislation No. 11.794/08. Animal care experimental procedures were previously approved by the Institutional Ethics Committee for Experimental Animals (No. 1389/15) on 18 November 2016.

### 5.6. Stereotaxic Surgery and Electrographic Recording

The animals were anesthetized with a mixture (1.3 mg/kg) of 10% ketamine and 2% xylazine (i.p.) and fixed in a stereotaxic apparatus. The intrahippocampal coordinates of cannulas and electrodes were established according to the Paxinos and Watson Atlas [68]. The cannula was implanted in one side of the hippocampus (injected side) and the electrode in the other side. The system was anchored to the skull with jeweler screws and dental acrylate. After surgery, the animals were housed individually and allowed to recover for a period of 4–5 days.

To evaluate the behavioral and electrophysiological effect induced by Tb II-I, the animals were individually placed in acrylate boxes (30 × 20 × 30 cm) inside of a Faraday cage. The intracerebral electrodes were connected to a data acquisition system (BIOPAC System Inc., Model MP150, Goleta, CA, USA), and the cerebral activity and the behavior were observed for 30 min (basal activity). Afterwards, Tb II-I (2 µg/2 µL, *n* = 6) or Ringer’s solution (2 µL; *n* = 6) were injected in the hippocampus using a 30-G needle connected to a 5-µL syringe in a slow flux (0.25 µL/s), and an uninterrupted observation and recording were performed for 4 successive hours.

Isolated or clustered spikes and moderate and/or intense discharges were considered epileptiform activity. The behavioral parameters such as salivary and lachrymal secretions, in which animals present an increase in the production of fluids in mouth and eyes, WDS, characterized by shakes in the body, penile erection, and myoclonus, characterized by muscular spasms in the body, were observed for later comparison between animals of the control group and the experimental group. Similar symptoms are also caused by other scorpion species such as *T. serrulatus* [69], *Androctonus* and *Buthus* [70].

### 5.7. Histological Analysis

One week after the experiment described above, the animals were anesthetized with carbon dioxide (CO_2_) and perfused via cardiac puncture with phosphate-buffered saline (PBS) and 10% buffered formalin. After decapitation, the brains were removed and stored in formalin for 1 week and then processed (successive alcohol baths ending with xylol overnight) and embedded in Paraplast^®^ (manufactured by Oxford Labware, St. Louis, MO, USA). The tissue was sliced (10 µm) and stained with cresyl violet. The number of cells in the CA1, CA3 and CA4 hippocampal areas was evaluated by light microscopy. Only pyramidal neurons with a visible nucleus and nucleolus were considered intact.

### 5.8. Cytokine Quantification

For cytokine quantification, 3 groups of animals (6 animals in each group) were used. After the stereotaxic surgery (as described above) and a 7-day recovery period, the first group received an intrahippocampal injection of 2 µg/2 µL of Tb II-I; the second one received an intrahippocampal injection of 2 µL Ringer’s solution; and the last one received an intrahippocampal injection of 10 µg/µL of LPS. Six hours after the injection, the animals were deeply anesthetized with carbon dioxide, and their brains were quickly removed and the hippocampi rapidly dissected.

All the procedures were performed on an ice pack. The tissue samples were immediately placed in a protease inhibitor solution (protease inhibitor cocktail Sigma P8340—Sigma—Aldrich, St. Louis, MO, USA), macerated in a Polytron^®^ (Kinematica AG Littau, Luzern, Switzerland) tissue homogenizer and centrifuged for 10 min (6707× *g*, 4 °C). The supernatant was collected and kept at −80 °C until the assay.

The cytokines IL-1α, IL-1β, IL-10, IL-6, IFN-γ and TNF- α were assayed by a sandwich enzyme-linked immunosorbent assay (ELISA), according to the manufacturer of the kit (IBL International GMBH, Hamburg, Germany).

## 6. Statistical Analysis

Data from behavioral and electrographic observation were analyzed by Fisher’s test (*p* < 0.05). Data from histological evaluation were analyzed by Student’s *t*-test (*p* < 0.05), and data from cytokine quantification were analyzed by ANOVA followed by Tukey’s test (*p* < 0.05). The GraphPad Prism 5.01 statistical program (GraphPad Software, La Jolla, CA, USA, 2007) was used. The tests were accepted as the most appropriate by the statistical program used for data analysis.

## Figures and Tables

**Table 1:** Behavioral and electrographic alterations observed after intrahippocampal injection of Fractions I, II, III and IV (2 µg/µL) or Ringer’s solution (1 µL, control group). WDS, wet dog shakes.

Alteration Observed	Fraction I *n* = 6	Fraction II *n* = 6	Fraction III *n* = 6	Fraction IV *n* = 6	Ringer *n* = 6
Prostration	33%	33%	17%	17%	0%
Respiratory difficulty	17%	50%	0%	0%	0%
Locomotor difficulty	0%	50%	0%	0%	0%
Salivary and lachrymal Secretions	17%	33%	0%	0%	0%
Myoclonus	0%	83%	0%	0%	0%
WDS	33%	83% *	0%	0%	0%
Spikes	33%	83% *	0%	0%	0%
Epileptiform discharges	33%	83% *	0%	0%	0%

The statistical analysis was performed using Fisher’s exact test, * *p* < 0.05.

**Table 2:** Theoretical and experimental masses of toxins present in Tb II-I.

Toxin	Theoretical Mass	Experimental Mass
Tbah02791/Toxin Tb4	6851.04	6851.71 ± 0.91
Tbah02765/Tb2 II	6950.03	6950.30 ± 0.98

**Table 3:** Behavioral and electrographic alterations observed after intrahippocampal injection of Tb II-I (2 µg/2 µL) or Ringer’s solution (2 µL, control group).

Treatment	N	Salivary and Lachrymal Secretions	WDS	Penile Erection	Myoclonus	Slow Waves	Grouped Spikes	Strong Discharges
Tb II-I	6	33%	67% *	50%	33%	33%	67% *	67% *
Ringer	6	0%	0%	0%	0%	0%	0%	0%

The statistical analysis was performed using Fisher’s exact test, * *p* < 0.05.

## Supplementary Materials

The following are available online at http://www.mdpi.com/2072-6651/10/6/250/s1: Figure S1: MALDI-TOF/MS profile of the Tb II-I, Tb II–II and Tb II-III fractions. (A) It was possible to observe an intense peak around 6800–6900 *m*/*z* in all three fractions. Besides, the ion 3437 in Fractions Tb II–II and Tb II–III is the double-charged ion [M + 2H] ^2+^ of 6870 *m*/*z*. (B) After limiting the mass range between 6750 and 6950 *m*/*z*, it was possible to observe the presence of 2–3 peaks in Tb II–II and Tb II–III, respectively, with middle to higher intensity. Instead of this, only Tb II–I shows one intensity peak at this mass range. Figure S2: Tryptic peptides identified for the Tbah02791 (toxin Tb4) protein. (A) Annotated spectrum with alignment of the ion *m/z* [575.77]^2+^ and its (B) ion table and error map. (C) Annotated spectrum with alignment of the ion *m/z* [652.89]^3+^ and its (D) ion table and error map. (E) Annotated spectrum with alignment of the ion *m/z* [1129.48]^3+^ and its (F) ion table and error map. Figure S3: Tryptic peptides identified for the Tbah02765 (toxin Tb2 II) protein. (A) Annotated spectrum with alignment of the ion *m/z* [667.62]^3+^ and its (B) ion table and error map. (C) Annotated spectrum with alignment of the ion *m/z* [1205.935]^2+^ and its (D) ion table and error map. (E) Annotated spectrum with alignment of the ion *m/z* [996.509]^1+^ and its (F) ion table and error map. Figure S4: Chymotryptic peptides identified for the Tbah02791 (toxin Tb4) and Tbah02765 (toxin Tb2 II) proteins. (A) Annotated spectrum with alignment of the ion *m/z* [586.244]^2+^ and its (B) ion table and error map identified for Tbah02791. (C) Annotated spectrum with alignment of the ion *m/z* [1086.938]^2+^ and its (D) ion table and error map identified for Tbah02765. (E) Annotated spectrum with alignment of the ion *m/z* [927.30]^1+^ and its (F) ion table and error map identified in both proteins. Figure S5: Chemical cleavage peptide identified for the Tbah02791 (toxin Tb4) protein. (A) Annotated spectrum with alignment of the ion *m/z* [734.303]^2+^ and its (B) ion table and error map identified. Figure S6: List of peptides identified for the Tbah02791 (toxin Tb4) and Tbah02765 (toxin Tb2 II) proteins.
