# Supplementary material for: Tb II-I, a Fraction Isolated from Tityus bahiensis Scorpion Venom, Alters Cytokines’: Level and Induces Seizures When Intrahippocampally Injected in Rats
Source: Toxins (Basel). 2018 Jun 19;10(6):250. doi: 10.3390/toxins10060250 (PMC6024361; doi:10.3390/toxins10060250)
Supplement: Supplementary file 1 [file toxins-10-00250-s001.pdf]

Supplementary Materials: Tb II-I, a fraction isolated from *Tityus bahiensis* scorpion venom, alters cytokines level and induces seizures when intrahippocampally injected in rats

Emidio Beraldo Neto, Douglas O. C. Mariano, Lucas A. Freitas, Ana L. C. Dorce, Adriana N. Martins, Daniel C. Pimenta, Fernanda C. V. Portaro, Daniela C. Carvalho, Valquiria A. C. Dorce and Ana L. A. Nencioni

A

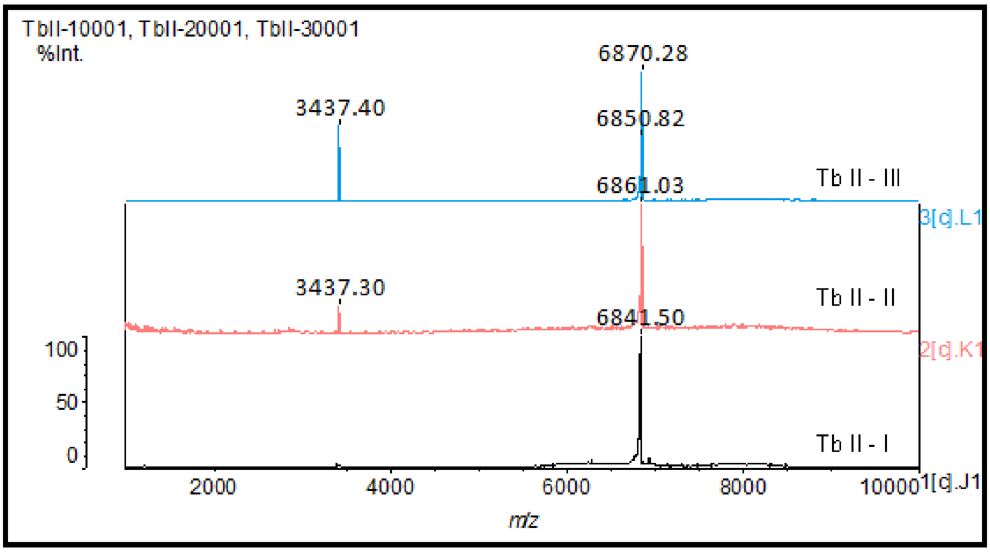

B

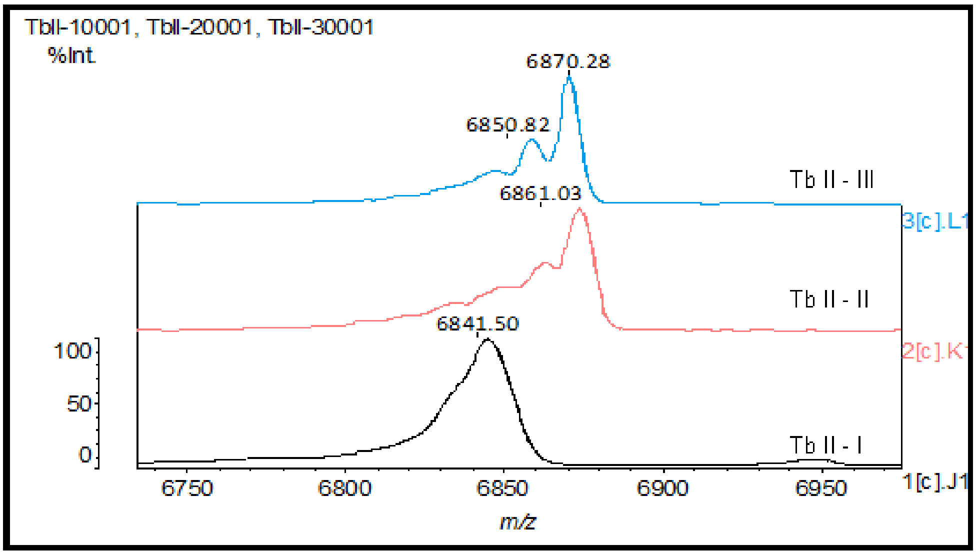

Figure S1. MALDI-TOF/MS profile of Tb II-I, Tb II–II and Tb II-III fractions. (**A**) It was possible to observe an intense peak around 6800 – 6900 m/z in all three fractions. Besides, the ion 3437 in fraction Tb II–II and Tb II–III is the double charged ion $[M + 2H]^{2+}$ of 6870 m/z. (**B**) After to limit the mass range between 6750 – 6950 m/z, it was possible to observe the presence of 2 – 3 peaks in Tb II–II and Tb II–III, respectively, with middle to higher intensity. Instead of it, only Tb II–I shows one intensity peak at this mass range.

A

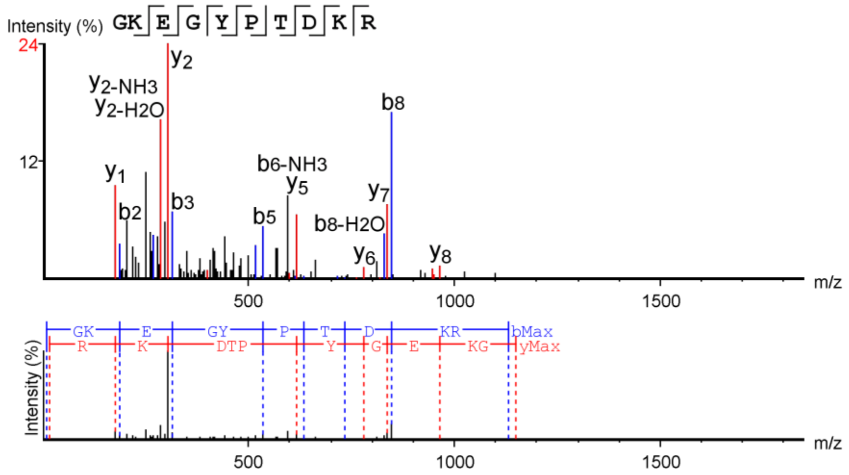

B

#	b	b-H2O	b-NH3	b (2+)	Seq	y	y-H2O	y-NH3	y (2+)	#
1	58.03	40.02	41.00	29.51	G					10
2	186.14	168.11	169.10	93.56	K	1093.56	1075.55	1076.54	547.28	9
3	315.16	297.16	298.14	158.08	E	965.47	947.50	948.43	483.23	8
4	372.19	354.18	355.16	186.59	G	836.42	818.38	819.46	418.71	7
5	535.27	517.22	518.23	268.19	Y	779.39	761.49	762.38	390.20	6
6	632.28	614.24	615.33	316.65	P	616.34	598.35	599.34	308.67	5
7	733.27	715.34	716.27	367.18	T	519.29	501.28	502.26	260.14	4
8	848.37	830.39	831.35	424.69	D	418.24	400.23	401.20	209.62	3
9	976.47	958.46	959.45	488.74	K	303.22	285.21	286.19	152.11	2
10					R	175.13	157.11	158.09	88.06	1

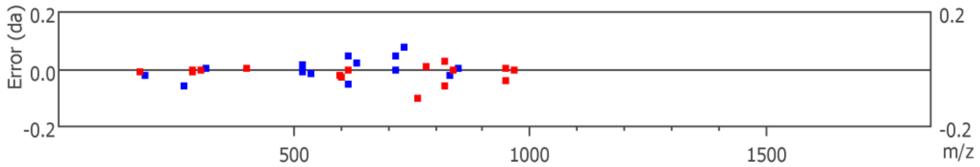

C

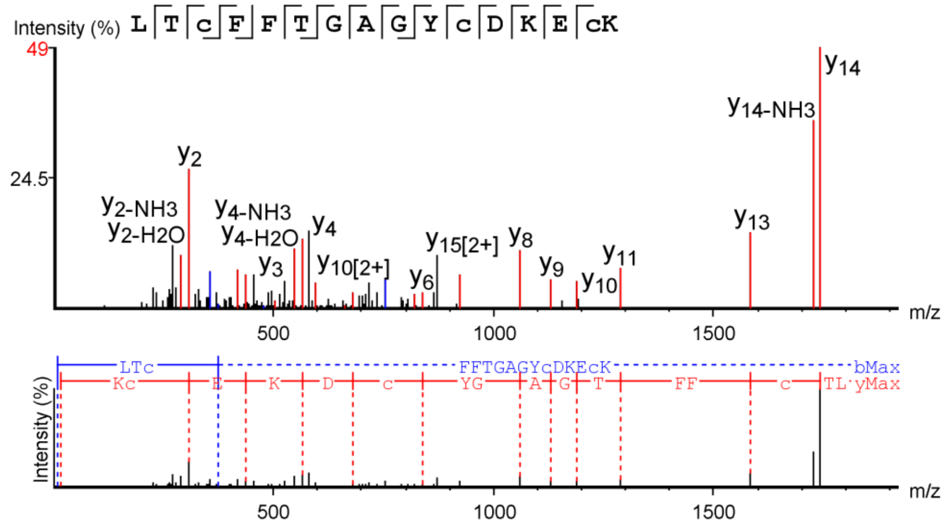

D

#	b	b-H2O	b-NH3	b (2+)	Seq	y	y-H2O	y-NH3	y (2+)	#
1	114.09	96.08	97.06	57.55	L					16
2	215.14	197.13	198.11	108.07	T	1843.74	1825.73	1826.72	922.37	15
3	375.17	357.19	358.14	188.09	C(+57.02)	1742.70	1724.69	1725.69	871.85	14
4	522.24	504.15	505.21	261.62	F	1582.66	1564.66	1565.64	791.83	13
5	669.31	651.30	652.28	335.15	F	1435.60	1417.59	1418.57	718.30	12
6	770.35	752.35	753.27	385.68	T	1288.49	1270.52	1271.50	644.76	11
7	827.38	809.37	810.35	414.19	G	1187.47	1169.47	1170.45	594.24	10
8	898.41	880.40	881.39	449.71	A	1130.44	1112.45	1113.43	565.73	9
9	955.43	937.42	938.41	478.21	G	1059.37	1041.41	1042.40	530.21	8
10	1118.50	1100.49	1101.47	559.75	Y	1002.40	984.39	985.37	501.71	7
11	1278.53	1260.52	1261.50	639.76	C(+57.02)	839.34	821.37	822.31	420.12	6
12	1393.56	1375.55	1376.53	697.28	D	679.29	661.29	662.28	340.15	5
13	1521.65	1503.64	1504.62	761.33	K	564.27	546.23	547.12	282.64	4
14	1650.69	1632.68	1633.67	825.85	E	436.24	418.17	419.17	218.59	3
15	1810.72	1792.71	1793.70	905.86	C(+57.02)	307.12	289.13	290.02	154.07	2
16					K	147.11	129.10	130.09	74.06	1

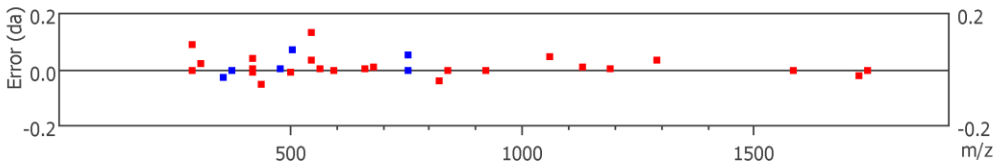

E

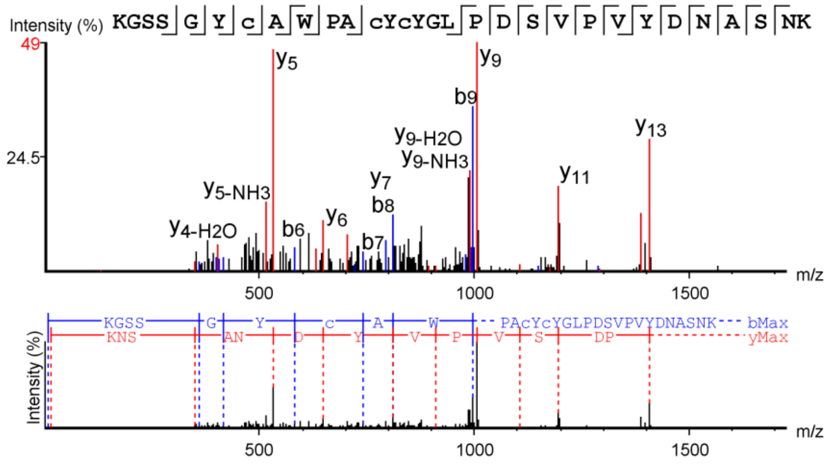

F

#	b	b-H2O	b-NH3	b (2+)	Seq	y	y-H2O	y-NH3	y (2+)	#
1	129.10	111.09	112.08	65.05	K					30
2	186.12	168.11	169.10	93.56	G	3258.36	3240.35	3241.33	1629.68	29
3	273.16	255.15	256.13	137.08	S	3201.34	3183.33	3184.31	1601.17	28
4	360.14	342.18	343.16	180.59	S	3114.31	3096.30	3097.28	1557.65	27
5	417.17	399.20	400.18	209.10	G	3027.27	3009.26	3010.25	1514.14	26
6	580.25	562.26	563.25	290.64	Y	2970.25	2952.24	2953.23	1485.63	25
7	740.33	722.33	723.28	370.65	C(+57.02)	2807.19	2789.18	2790.16	1404.09	24
8	811.35	793.31	794.33	406.16	A	2647.16	2629.15	2630.13	1324.08	23
9	997.42	979.45	980.36	499.21	W	2576.12	2558.11	2559.09	1288.58	22
10	1094.47	1076.46	1077.45	547.74	P	2390.04	2372.03	2373.02	1195.52	21
11	1165.51	1147.46	1148.47	583.26	A	2292.99	2274.98	2275.96	1146.99	20
12	1325.54	1307.53	1308.51	663.27	C(+57.02)	2221.95	2203.94	2204.93	1111.48	19
13	1488.60	1470.59	1471.58	744.80	Y	2061.92	2043.91	2044.90	1031.46	18
14	1648.63	1630.62	1631.61	824.82	C(+57.02)	1898.86	1880.85	1881.83	949.93	17
15	1811.70	1793.69	1794.67	906.35	Y	1738.83	1720.82	1721.80	869.91	16
16	1868.72	1850.71	1851.69	934.86	G	1575.76	1557.75	1558.74	788.38	15
17	1981.80	1963.79	1964.78	991.43	L	1518.74	1500.73	1501.72	759.87	14
18	2078.86	2060.85	2061.83	1039.93	P	1405.66	1387.64	1388.64	703.30	13
19	2193.88	2175.87	2176.86	1097.44	D	1308.61	1290.60	1291.50	654.80	12
20	2280.92	2262.90	2263.89	1140.96	S	1193.60	1175.56	1176.55	597.29	11
21	2379.98	2361.97	2362.96	1190.49	V	1106.47	1088.54	1089.52	553.77	10
22	2477.04	2459.03	2460.01	1239.02	P	1007.47	989.47	990.46	504.24	9
23	2576.10	2558.09	2559.08	1288.58	V	910.37	892.37	893.40	455.71	8
24	2739.17	2721.16	2722.14	1370.08	Y	811.35	793.31	794.33	406.16	7
25	2854.20	2836.18	2837.17	1427.60	D	648.28	630.22	631.28	324.65	6
26	2968.24	2950.23	2951.21	1484.62	N	533.26	515.26	516.23	267.13	5
27	3039.28	3021.26	3022.25	1520.14	A	419.22	401.15	402.20	210.11	4
28	3126.31	3108.30	3109.28	1563.65	S	348.21	330.18	331.16	174.59	3
29	3240.35	3222.34	3223.32	1620.68	N	261.16	243.15	244.13	131.22	2
30					K	147.11	129.10	130.09	74.06	1

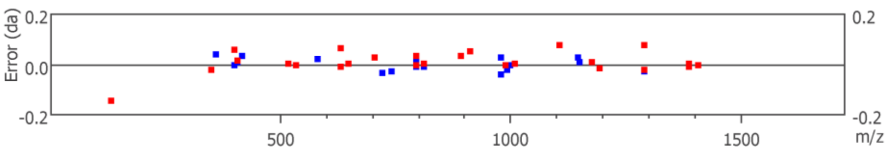

Figure S2. Tryptic peptides identified for Tbah02791 (Toxin Tb4) protein. (**A**) Annotated spectrum with alignment of the ion 575.77 2+ and its (**B**) ion table and error map. (**C**) Annotated spectrum with alignment of the ion 652.89 3+ and its (**D**) ion table and error map. (**E**) Annotated spectrum with alignment of the ion 1129.48 3+ and its (**F**) ion table and error map.

A

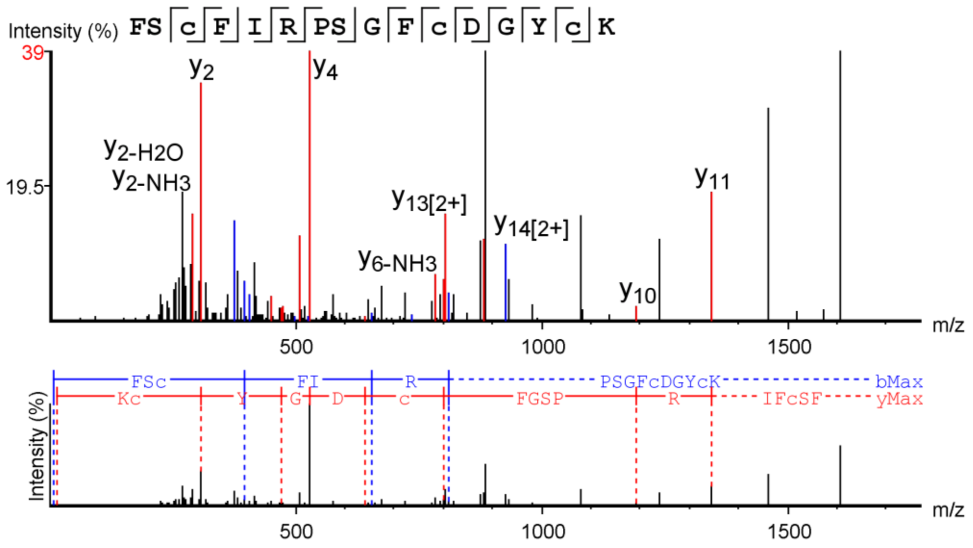

B

#	b	b-H2O	b-NH3	b (2+)	Seq	y	y-H2O	y-NH3	y (2+)	#
1	148.08	130.07	131.05	74.54	F					16
2	235.11	217.10	218.08	118.05	S	1853.78	1835.77	1836.75	927.39	15
3	395.16	377.13	378.11	198.07	C(+57.02)	1766.74	1748.73	1749.72	883.92	14
4	542.21	524.17	525.18	271.60	F	1606.71	1588.70	1589.69	803.92	13
5	655.25	637.28	638.26	328.15	I	1459.65	1441.64	1442.62	730.32	12
6	811.35	793.38	794.37	406.24	R	1346.62	1328.55	1329.53	673.78	11
7	908.45	890.43	891.42	454.72	P	1190.40	1172.45	1173.43	595.73	10
8	995.48	977.47	978.45	498.21	S	1093.41	1075.40	1076.38	547.20	9
9	1052.50	1034.49	1035.47	526.75	G	1006.38	988.37	989.35	503.69	8
10	1199.57	1181.56	1182.54	600.28	F	949.35	931.34	932.33	475.22	7
11	1359.60	1341.59	1342.57	680.30	C(+57.02)	802.29	784.28	785.27	401.64	6
12	1474.62	1456.61	1457.60	737.81	D	642.25	624.24	625.23	321.63	5
13	1531.65	1513.64	1514.62	766.32	G	527.23	509.17	510.20	264.11	4
14	1694.71	1676.70	1677.68	847.85	Y	470.22	452.15	453.18	235.60	3
15	1854.74	1836.73	1837.71	927.87	C(+57.02)	307.15	289.15	290.10	154.07	2
16					K	147.11	129.10	130.09	74.06	1

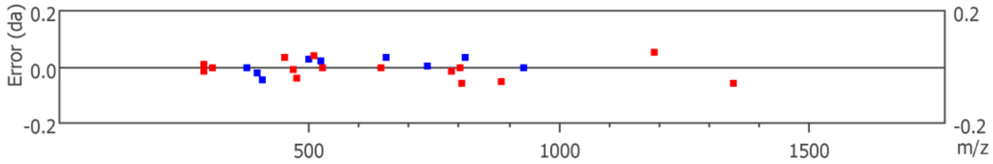

C

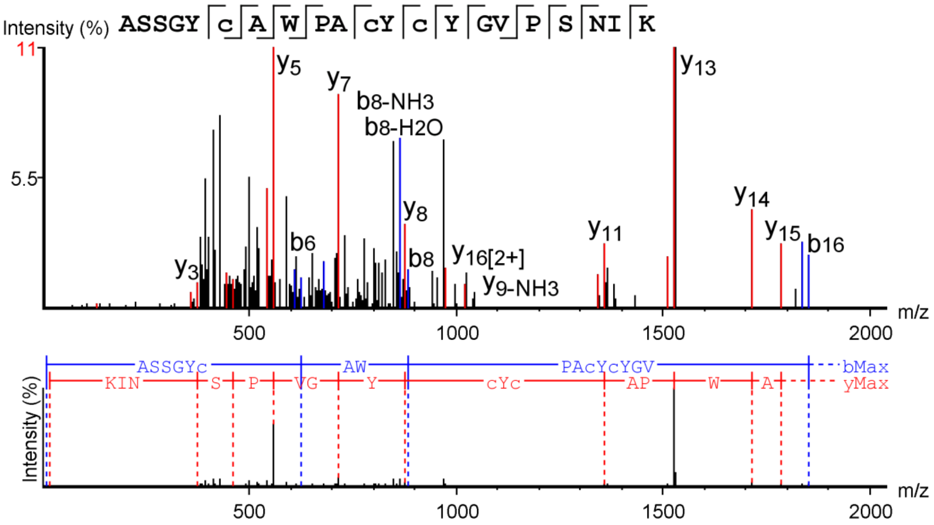

D

#	b	b-H2O	b-NH3	b (2+)	Seq	y	y-H2O	y-NH3	y (2+)	#
1	72.04	54.03	55.02	36.52	A					21
2	159.08	141.07	142.05	80.04	S	2339.99	2321.98	2322.96	1170.49	20
3	246.11	228.10	229.08	123.55	S	2252.96	2234.95	2235.93	1126.98	19
4	303.13	285.12	286.10	152.07	G	2165.92	2147.91	2148.90	1083.46	18
5	466.19	448.18	449.17	233.60	Y	2108.90	2090.89	2091.88	1054.95	17
6	626.20	608.21	609.20	313.61	C(+57.02)	1945.84	1927.83	1928.81	973.37	16
7	697.26	679.22	680.23	349.13	A	1785.82	1767.80	1768.78	893.40	15
8	883.32	865.32	866.32	442.17	W	1714.75	1696.76	1697.74	857.89	14
9	980.39	962.38	963.37	490.70	P	1528.69	1510.76	1511.67	764.85	13
10	1051.43	1033.42	1034.40	526.22	A	1431.64	1413.63	1414.61	716.32	12
11	1211.46	1193.45	1194.43	606.23	C(+57.02)	1360.63	1342.59	1343.54	680.80	11
12	1374.52	1356.51	1357.50	687.76	Y	1200.57	1182.56	1183.54	600.79	10
13	1534.56	1516.54	1517.53	767.78	C(+57.02)	1037.51	1019.50	1020.47	519.25	9
14	1697.62	1679.61	1680.59	849.31	Y	877.47	859.47	860.45	439.24	8
15	1754.64	1736.63	1737.61	877.82	G	714.41	696.40	697.39	357.71	7
16	1853.79	1835.71	1836.71	927.35	V	657.39	639.38	640.37	329.20	6
17	1950.76	1932.75	1933.73	975.88	P	558.33	540.31	541.32	279.66	5
18	2037.79	2019.78	2020.77	1019.40	S	461.19	443.26	444.24	231.14	4
19	2151.84	2133.83	2134.81	1076.42	N	374.26	356.23	357.25	187.62	3
20	2264.92	2246.91	2247.89	1132.96	I	260.20	242.19	243.17	130.60	2
21					K	147.11	129.10	130.21	74.06	1

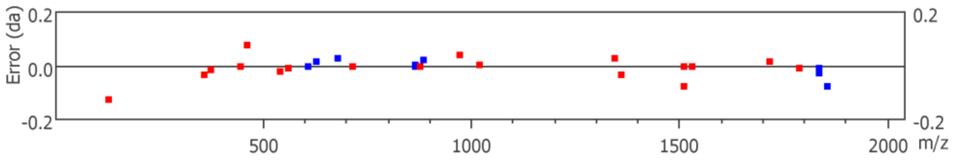

E

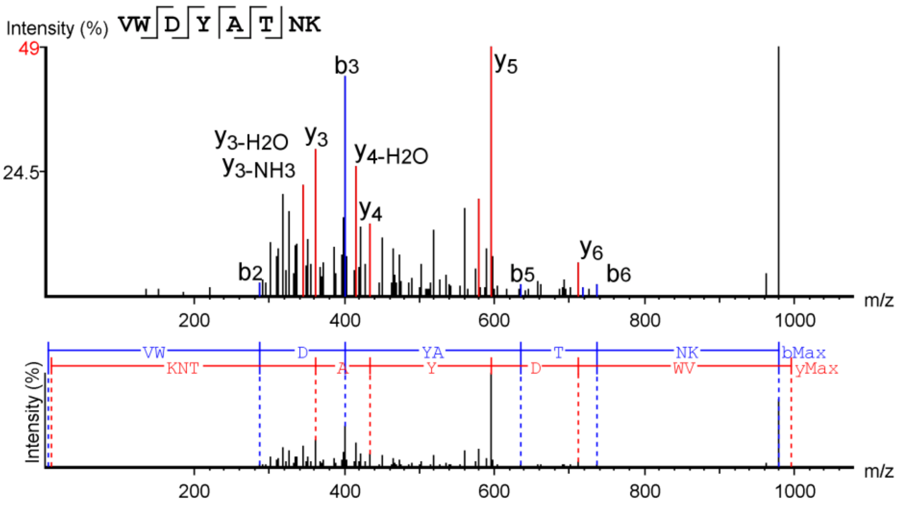

F

#	b	b-H2O	b-NH3	b (2+)	Seq	y	y-H2O	y-NH3	y (2+)	#
1	100.08	82.07	83.05	50.54	V					8
2	286.14	268.14	269.13	143.58	W	897.41	879.40	880.38	449.21	7
3	401.18	383.17	384.16	201.09	D	711.29	693.32	694.30	356.17	6
4	564.25	546.24	547.22	282.62	Y	596.32	578.29	579.29	298.65	5
5	635.31	617.27	618.26	318.14	A	433.17	415.15	416.21	217.12	4
6	736.30	718.38	719.30	368.67	T	362.16	344.17	345.21	181.60	3
7	850.37	832.36	833.35	425.69	N	261.16	243.15	244.13	131.08	2
8					K	147.11	129.10	130.09	74.06	1

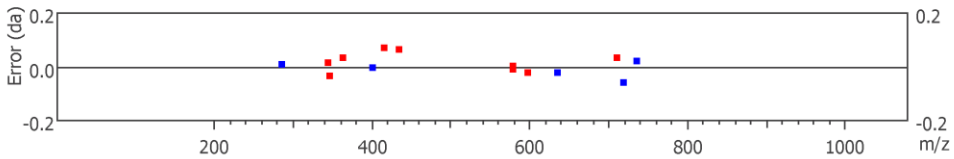

Figure S3. Tryptic peptides identified for Tbah02765 (Toxin Tb2 II) protein. (**A**) Annotated spectrum with alignment of the ion 667.62 3+ and its (**B**) ion table and error map. (**C**) Annotated spectrum with alignment of the ion 1205.935 2+ and its (**D**) ion table and error map. (**E**) Annotated spectrum with alignment of the ion 996.509 1+ and its (**F**) ion table and error map.

A

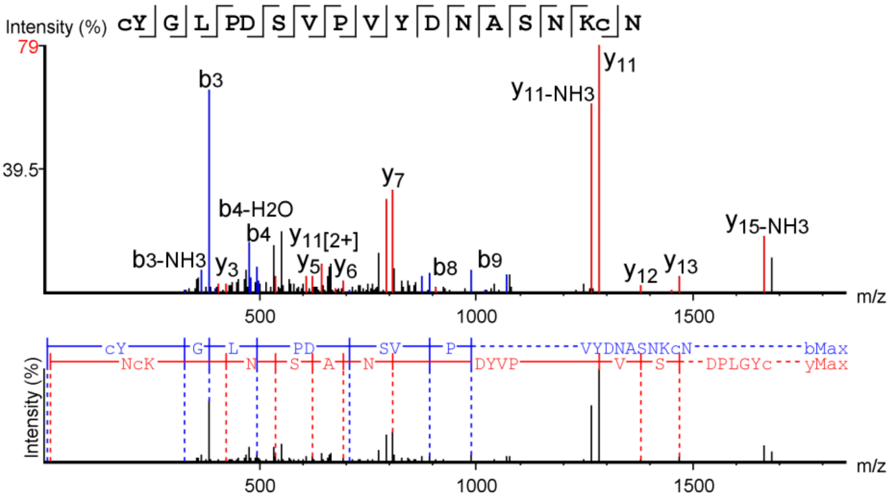

B

#	b	b-H2O	b-NH3	b (2+)	Seq	y	y-H2O	y-NH3	y (2+)	#
1	161.04	143.03	144.01	81.02	C(+57.02)					19
2	324.02	306.09	307.07	162.55	Y	2012.90	1994.89	1995.87	1006.95	18
3	381.12	363.11	364.11	191.06	G	1849.84	1831.83	1832.81	925.42	17
4	494.19	476.21	477.15	247.60	L	1792.82	1774.81	1775.79	896.91	16
5	591.26	573.25	574.23	296.13	P	1679.73	1661.72	1662.67	840.37	15
6	706.21	688.28	689.26	353.64	D	1582.68	1564.67	1565.65	791.84	14
7	793.32	775.31	776.29	397.08	S	1467.60	1449.64	1450.58	734.33	13
8	892.36	874.33	875.37	446.69	V	1380.61	1362.61	1363.59	690.81	12
9	989.45	971.43	972.41	495.25	P	1281.52	1263.54	1264.49	641.25	11
10	1088.51	1070.41	1071.48	544.75	V	1184.50	1166.49	1167.47	592.75	10
11	1251.57	1233.56	1234.54	626.29	Y	1085.43	1067.42	1068.40	543.22	9
12	1366.60	1348.59	1349.57	683.80	D	922.37	904.36	905.36	461.68	8
13	1480.64	1462.63	1463.61	740.82	N	807.32	789.33	790.30	404.20	7
14	1551.68	1533.67	1534.65	776.34	A	693.29	675.29	676.22	347.15	6
15	1638.71	1620.70	1621.68	819.86	S	622.26	604.25	605.21	311.63	5
16	1752.75	1734.74	1735.73	876.88	N	535.21	517.22	518.20	268.11	4
17	1880.85	1862.84	1863.82	940.92	K	421.15	403.16	404.20	211.09	3
18	2040.88	2022.87	2023.85	1020.88	C(+57.02)	293.09	275.08	276.06	147.05	2
19					N	133.06	115.05	116.03	67.03	1

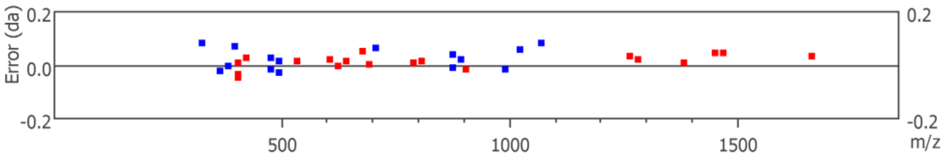

C

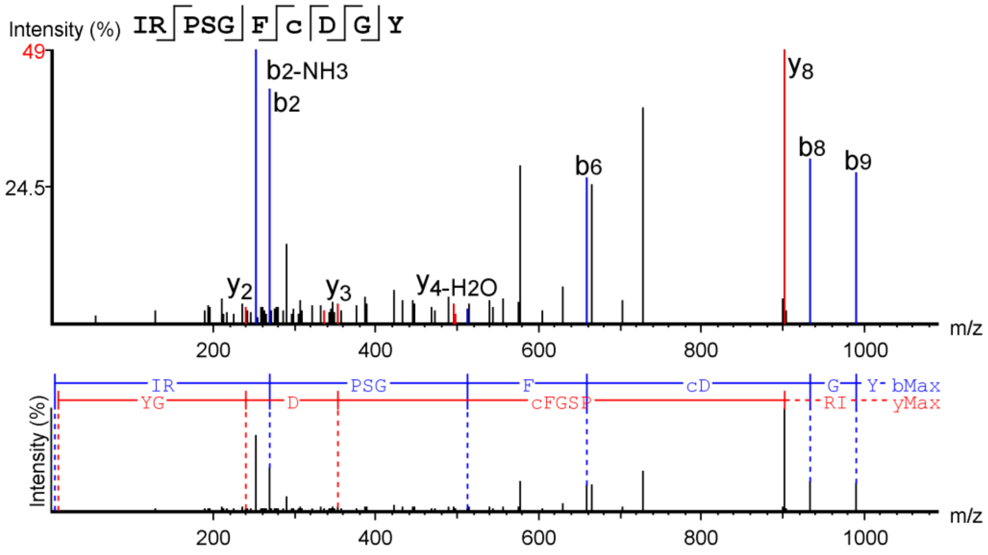

D

#	b	b-H2O	b-NH3	b (2+)	Seq	y	y-H2O	y-NH3	y (2+)	#
1	114.09	96.08	97.06	57.55	I					10
2	270.20	252.18	253.12	135.60	R	1058.44	1040.43	1041.41	529.72	9
3	367.25	349.24	350.22	184.12	P	902.32	884.32	885.31	451.67	8
4	454.28	436.27	437.25	227.64	S	805.28	787.27	788.26	403.14	7
5	511.43	493.29	494.27	256.15	G	718.25	700.24	701.22	359.63	6
6	658.32	640.36	641.34	329.68	F	661.23	643.22	644.20	331.11	5
7	818.40	800.39	801.37	409.70	C(+57.02)	514.16	496.16	497.15	257.58	4
8	933.35	915.41	916.40	467.21	D	354.07	336.12	337.10	177.56	3
9	990.43	972.44	973.42	495.72	G	239.05	221.09	222.08	120.05	2
10					Y	182.08	164.07	165.05	91.54	1

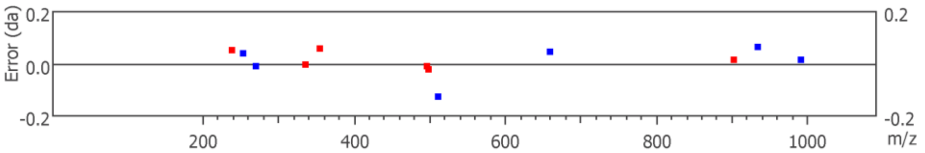

E

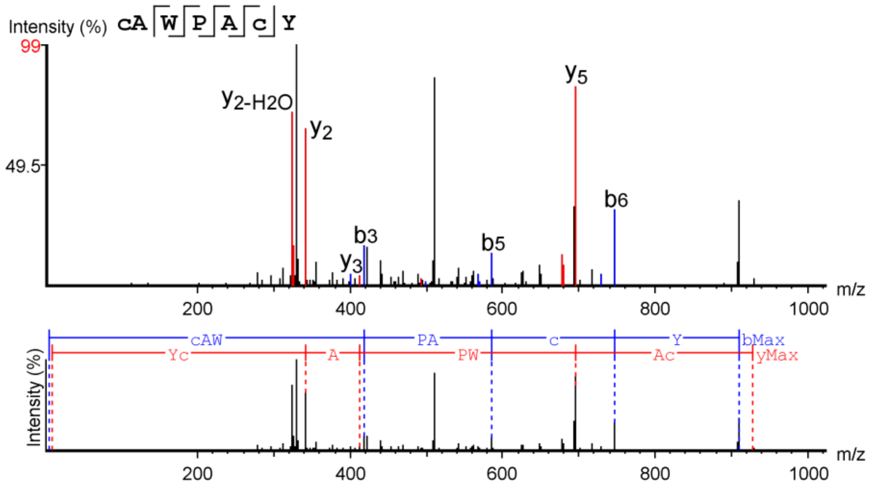

F

#	b	b-H2O	b-NH3	b (2+)	Seq	y	y-H2O	y-NH3	y (2+)	#
1	161.04	143.03	144.01	81.02	C(+57.02)					7
2	232.08	214.07	215.05	116.54	A	767.32	749.31	750.29	384.16	6
3	418.09	400.17	401.17	209.58	W	696.25	678.21	679.23	348.64	5
4	515.21	497.20	498.14	258.10	P	510.20	492.11	493.17	255.60	4
5	586.18	568.21	569.23	293.62	A	413.14	395.14	396.12	207.07	3
6	746.24	728.23	729.25	373.64	C(+57.02)	342.07	324.08	325.14	171.56	2
7					Y	182.08	164.07	165.05	91.54	1

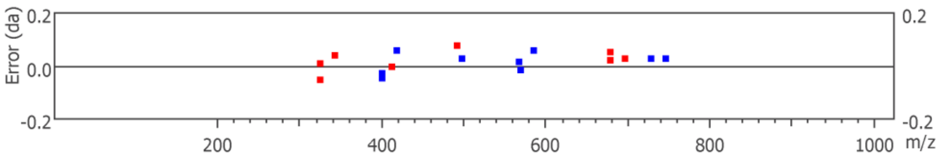

Figure S4. Chymotryptic peptides identified for Tbah02791 (Toxin Tb4) and Tbah02765 (Toxin Tb2 II) proteins. (**A**) Annotated spectrum with alignment of the ion 586.244 2+ and its (**B**) ion table and error map identified for Tbah02791. (**C**) Annotated spectrum with alignment of the ion 1086.938 2+ and its (**D**) ion table and error map identified for Tbah02765. (**E**) Annotated spectrum with alignment of the ion 927.30 1+ and its (**F**) ion table and error map identified in both proteins.

A

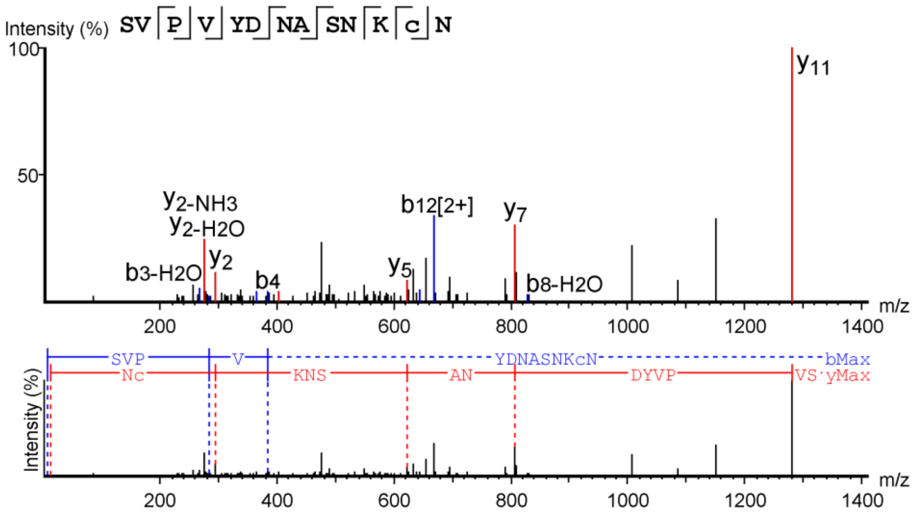

B

#	b	b-H2O	b-NH3	b (2+)	Seq	y	y-H2O	y-NH3	y (2+)	#
1	88.04	70.03	71.01	44.52	S					13
2	187.11	169.10	170.08	94.05	V	1380.62	1362.61	1363.59	690.81	12
3	284.11	266.06	267.13	142.58	P	1281.58	1263.54	1264.53	641.28	11
4	383.26	365.23	366.20	192.11	V	1184.50	1166.49	1167.47	592.75	10
5	546.29	528.28	529.27	273.65	Y	1085.43	1067.42	1068.40	543.22	9
6	661.32	643.33	644.29	331.16	D	922.37	904.36	905.34	461.68	8
7	775.36	757.35	758.34	388.18	N	807.35	789.33	790.31	404.17	7
8	846.40	828.32	829.37	423.70	A	693.30	675.29	676.27	347.15	6
9	933.43	915.42	916.40	467.22	S	622.26	604.25	605.23	311.63	5
10	1047.47	1029.46	1030.45	524.24	N	535.23	517.22	518.20	268.11	4
11	1175.57	1157.56	1158.54	588.28	K	421.19	403.19	404.16	211.09	3
12	1335.60	1317.59	1318.57	668.29	C(+57.02)	293.12	275.08	276.12	147.05	2
13					N	133.06	115.05	116.03	67.03	1

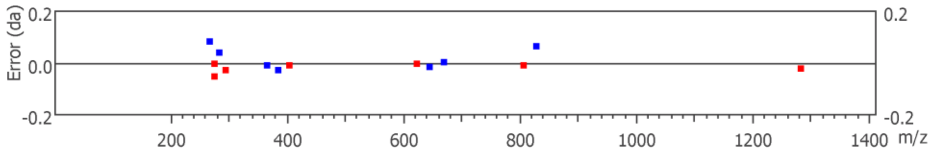

Figure S5. Chemical cleavage peptide identified for Tbah02791 (Toxin Tb4) protein. (**A**) Annotated spectrum with alignment of the ion 734.303 2+ and its (**B**) ion table and error map identified.

Table S1. List of peptides identified for Tbah02791 (Toxin Tb4) and Tbah02765 (Toxin Tb2 II) proteins.

Protein Accession	Cleavage	Peptide	-10lgP	Mass	Length	ppm	m/z	z
Tbah02791 (Toxin Tb4)	Trypsin	K.LTC(+57.02)*FFTGAGYC(+57.02)*DK.E	69.51	1538.65	13	-0.7	770.3334	2
		K.LTC(+57.02)*FFTGAGYC(+57.02)*DKEC(+57.02)*K.L	55.62	1955.82	16	-75.8	652.8984	3
		K.KGSSGYC(+57.02)*AWPAC(+57.02)*YC(+57.02)*YGLPDSVPVYDNASNK.C	42.75	3385.44	30	-6.6	1129.4824	3
		G.GKEGYPTDKR.G	37.63	1149.57	10	-37.7	575.7745	2
		K.EGYPTDKR.G	24.92	964.46	8	-36	483.2206	2
		G.GKEGYPTDK.R	22.39	993.47	9	-70.5	497.7106	2
	Chymotrypsin	Y.C(+57.02)*AWPAC(+57.02)*Y.C	22.48	926.34	7	-42.8	927.3091	1
		Y.C(+57.02)*YGLPDSVPVYDNASNKC(+57.02)*N.K	16.96	2171.92	19	-29.1	1086.9384	2
	Formic acid	T.SVPVYDNASNKC(+57.02)*N.A	20.32	1466.64	13	-36.1	734.3038	2
Tbah02765 (Toxin Tb2 II)	Trypsin	K.VWDYATNK.C	33.92	995.47	8	30.6	996.509	1
		K.FSC(+57.02)*FIRPSGFC(+57.02)*DGYC(+57.02)*K.T	29.13	1999.83	16	2.2	667.6214	3
		K.ASSGYC(+57.02)*AWPAC(+57.02)*YC(+57.02)*YGVPSNIK.V	21.78	2410.01	21	-66.8	1205.9358	2
	Chymotrypsin	F.IRPSGFC(+57.02)*DGY.C	23.3	1170.51	10	-33	586.2444	2
		Y.C(+57.02)*AWPAC(+57.02)*Y.C	22.48	926.34	7	-42.8	927.3091	1

* +57.02: Carbamidomethylation.
